# Cholera Precautions Suggested

**Published:** 1890-10

**Authors:** 


### Editorial, Etc.

CHOLERA PRECAUTIONS SUGGESTED.—The singular way in which the case of cholera reported from London was developed carries with it a warning which the quarantine officers in every port of this country would do well to heed and guard against. It seems that the patient on arriving at London from Calcutta put on some undergarments brought from India which he had not worn on the voyage. The germs of the cholera were in these garments, and though long dormant, soon displayed their activity when brought into the open air, This, at least, is the tenor of the New York Herald's advices, and the conclusion reached after thorough investigation of the case and interviewing the patient himself, who had circulated freely about London before he was taken to a hospital, and may have thus spread the germs of the disease. The physicians of the hospital entertained no doubt of the genuineness of the Asiatic type of the disease, but the government board later on concluded that although clinically undistinguishable from the symptoms of real cholera, the case may be local cholera, such as occurs in London every year. Nevertheless dispatches from the East continue to report the progress of real cholera, and it would be well for the health authorities to exercise even more of their habitual vigilance to prevent the introduction or spread in our ports by shipping.

